# The use of a novel toe–thumb pressure index for assessing arterial status in the lower limb. A reliability and validity study

**DOI:** 10.1002/jfa2.70011

**Published:** 2024-10-19

**Authors:** Juliana Mazzeo, Helen A. Banwell, Peta E. Tehan, Grace Anderson, Kristin Graham

**Affiliations:** ^1^ Allied Health and Human Performance Innovation, Implementation and Clinical Translation in Health (IIMPACT) University of South Australia Adelaide South Australia Australia; ^2^ School of Clinical and Molecular Sciences, Faculty of Medicine Nursing and Health School of Clinical Sciences Monash University Clayton Victoria Australia; ^3^ School of Health Sciences, College of Health Medicine and Wellbeing University of Newcastle New Castle New South Wales Australia; ^4^ Allied Health and Human Performance University of South Australia Adelaide South Australia Australia

**Keywords:** peripheral arterial disease, reliability study, thumb pressure, toe‐brachial index, validity study

## Abstract

**Aims:**

This study explored the reliability, validity and perceived comfort of a novel thumb pressure measure and calculation of a toe–thumb index to identify their suitability as an adjunct or alternatives to ankle–brachial and toe–brachial indices.

**Methods and Results:**

Repeated manual thumb and toe systolic blood pressures were conducted using two raters, over two time points, on 34 healthy participants. Concurrent automated toe, thumb and brachial systolic blood pressures as well as comfort ratings for these measures (using a 10 mm visual analogue scale) were captured once by a research assistant. Automated thumb and brachial measures showed fair correlation (*ρ* = 0.36, *p* = 0.03) and a toe–thumb index and toe–brachial index good correlation (*ρ* = 0.62, *p* < 0.01). Intraclass correlation coefficients (ICC) identified moderate intra‐rater reliability for manual thumb pressures for Rater 1 and 2 (ICC 0.57, 95% CI [0.14, 0.79] and ICC 0.74, 95% CI [0.49, 0.87], respectively), while inter‐rater reliability was poor (ICC = 0.16, 95% CI [−0.85, 0.47]). Concurrent validity comparing manual and automated measures for thumb pressure was also poor (ICC −0.05, 95% CI [−1.06, 0.72] and ICC 0.42, 95% CI [−0.16, 0.72] Rater 1 and 2 respectively). Thumb measures were significantly more comfortable than brachial measures (5 mm, *p* < 0.00).

**Conclusion:**

Thumb systolic pressures are correlated with brachial systolic pressures, with reasonable intra‐rater reliability, however, correlation is only fair and measurement error wider than clinically acceptable. Furthermore, manual measures are poorly correlated with automated units. Consequently, caution is required in applying these techniques. As thumb measures were perceived as significantly more comfortable than brachial measures and have an advantage where brachial pressures cannot, or should not, be obtained, further evaluation is warranted.

## 1 BACKGROUND

Peripheral arterial disease (PAD) is characterised by atherosclerotic lesions in blood vessels other than those supplying the heart and brain [1] with the lower extremities most frequently affected [1]. PAD affects over 200 million people globally [1, 2]. Risk factors for PAD include increasing age, male sex, increased body mass index (BMI), diabetes mellitus, smoking, hypertension and hypertriglyceridaemia [1, 3, 4]. Common symptoms of PAD include intermittent claudication (muscle pain with exercise) and ischaemic rest pain [3]. These symptoms can reduce participation in activities of daily living, physical activity and quality of life [5, 6]. Chronic limb threatening ischaemia is the end‐stage of PAD and can result in significantly increased risk of cardiovascular mortality, as well as lower limb ulceration, pain and lower limb amputation [7]. In Australia, the prevalence of PAD is 10%–15%, increasing to 22% in people aged over 75 years [8, 9] and incurs the highest health care costs and hospitalisation rates for all cardiovascular diseases [10]. Avoidable lower limb amputations are estimated to cost the public health system AUD $1.6 billion annually [11, 12]. Therefore, timely diagnosis of PAD is critical to facilitate appropriate management of risk factors and reduce complications. Non‐invasive methods are predominantly used to identify PAD which may include the ankle–brachial index (ABI), toe‐brachial index (TBI) or toe pressures alone [3, 13].

Global vascular guidelines recommend ABI measurements as the first‐line testing method to identify PAD [7, 14]. An ABI is calculated by dividing ankle systolic blood pressure by brachial systolic blood pressure [13]. ABI outcomes of <0.9 indicate potential PAD [15], and values exceeding 1.3 suggest the presence of medial arterial calcification [16], which is the deposition of bone morphogenic protein in the arterial wall leading to incompressibility [17]. The TBI is calculated by dividing toe systolic blood pressure by the highest of the brachial systolic blood pressures [16].

ABIs are highly variable in terms of intra‐ and inter‐rater reliability, with outcomes ranging from poor to excellent across different patient populations [18]. TBIs are reportedly more reliable, with fair to good intra‐rater reliability and excellent inter‐rater reliability observed [19], however, a wider range of measurement error was observed in individuals with diabetes [20]. Toe pressures have demonstrated excellent inter‐rater and intra‐rater reliability in people with and without diabetes with wide levels of agreement (LOA) demonstrated across both groups [21]. Both the ABI and TBI have demonstrated good validity for the detection of PAD, with TBIs being recommended to enhance diagnostic evaluation when ABIs are borderline or normal [22, 23]. This is particularly important when medial arterial calcification may be present, where atherosclerotic changes within arterial walls may inhibit the occlusion required to obtain the brachial and/or ankle blood pressures required for ABIs. Medial arterial calcification is more prevalent in populations most at risk of PAD, including when the person is older, is a smoker, has type 2 diabetes, increased cholesterol levels, or a higher BMI [17]. Toe pressures use digital arteries, where the smaller size of vessels and increased distance from the heart reduces the impact of medial artery calcification [16] but results in lower pressures than expected for brachial and ankle outcomes, potentially reducing identification of artery deficit [24]. TBI's compare this small vessel measure (toe pressure) to a large vessel measure (brachial pressure) extrapolating equivalence between the two.

Importantly, ABI and TBI calculations rely upon accurate measurement of brachial pressures, however, there are occasions where this is not possible. For example, when a person has lymphoedema, is post‐mastectomy and lymph node removal [25], or where arteriovenous fistulas [26], injuries, open wounds or infection exist [27]. Using a thumb pressure and a calculated thumb–toe index has the potential to reliably identify upper and lower limb blood pressure differences whilst negating these concerns. Using a thumb pressure measure over a brachial measure may also improve physical distancing between clinician and patient [28], and will be more comfortable in situations where physical touch or removal of clothing can cause distress [29].

The primary aims of this study were to determine if thumb pressures and a toe–thumb index could be a valid alternative to indices using brachial pressures in clinicians who have experience in conducting ABI and TBI measures. The secondary aims were to determine the concurrent validity of measures using manual and automated Doppler units as well as to compare perceived comfort of brachial and thumb blood pressure measurements.

## 2 METHODS

Criterion validity for thumb blood pressure measures and a ‘toe–thumb index’ was determined by comparisons with the reference standards of automated brachial and TBI measures respectively. Inter‐ and intra‐rater reliability was via a test–retest design seeking correlations between manual toe and thumb systolic blood pressure measures. Concurrent validity investigations compared automatic to manual Doppler units for the same novel measures. Participant preference was also explored. The study was developed in line with the guidelines for reporting reliability and agreement studies [30].

### 2.1 Raters

Two raters, a podiatrist in training (JM) and a podiatrist (HAB) conducted each manual measurement. Two research assistants (KG and GA) conducted the automated measurements. The podiatrist in training (JM) was a final year undergraduate student and had been trained in automated and manual measurements during the course of their degree. The podiatrist (HAB) had over 25 years of clinical practice, predominantly using automated measurements. Raters were involved in the development of the protocol and reviewed the final protocol. The protocol, including practice of manual measures, was piloted twice, once to allow open practice and discussion of measurements and once to ensure the protocol adequately allowed for participant blinding.

### 2.2 Participants

A convenience sample was recruited from the University of South Australia (UniSA) podiatry staff and student cohort. To be eligible for inclusion, participants need to be aged 18–44 years [31] and be non‐smokers. Exclusion criteria included diabetes, history of cardiovascular disease, BMI <18.5 or >30 [32], an allergy to ultrasound gel, scleroderma, Raynaud's disease or any inflammatory, musculoskeletal or neurological disorders that may have impacted blood pressure measurements. All participants gave written informed consent and were aware they could withdraw participation at any time. Ethics approval was obtained from the University of South Australia's Human Research Ethics Committee (Protocol number 204110).

### 2.3 Measures

**FIGURE 1:**
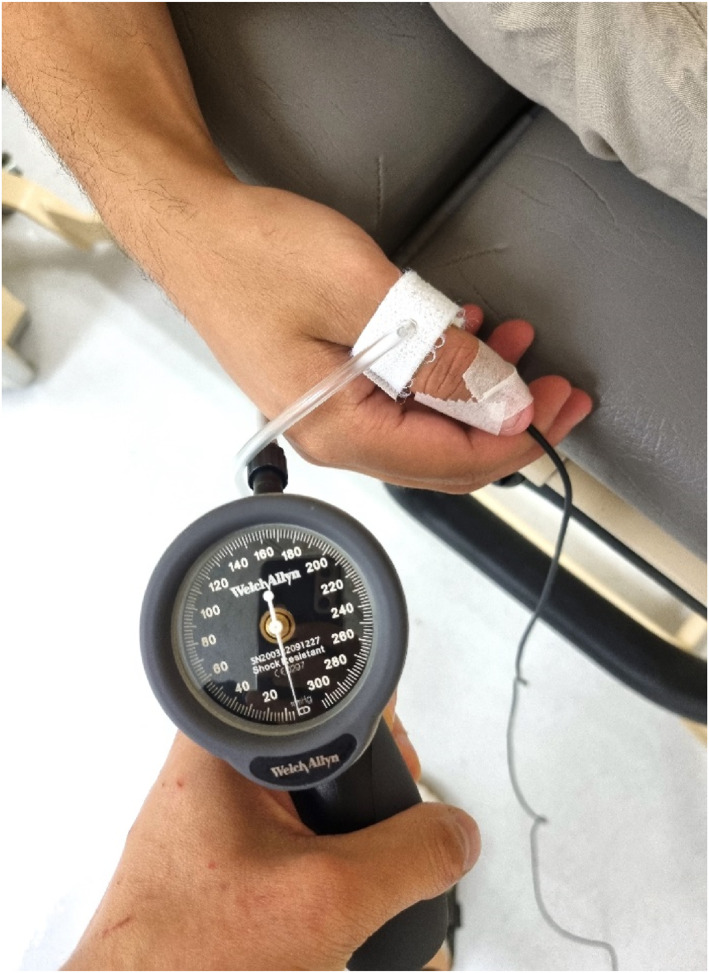
Manual thumb blood pressure with sphygmomanometer.

Manual toe and thumb pressures were taken with a handheld Hadeco ES100VX Mini Doppler (Hadeco). Automated brachial, toe and thumb pressures were taken with a Hadeco Smartdop 30EX (Hadeco). An 8 mmHz Doppler probe and standard 10 cm inflatable cuff were used to measure automated brachial blood pressures. Cuff size was 1.5 times the limb diameter in accordance with international guidelines [13]. Thumb and toe pressure measurements were collected using a Photoplethysmography (PPG) probe and digital pressure cuff of 2.5 × 12 cm for toes and 1.9 × 9 cm for thumbs. A sphygmomanometer was used for manual measures (Figure 1). The digital cuffs were placed at the proximal toe or thumb. The PPG probe was placed distally and secured with hypafix tape (Figure 1).

The novel toe–thumb index outcomes were calculated via dividing toe pressures over thumb pressures.

Comfort ratings were collected on participants perception of comfort for both brachial and thumb pressure readings via a 10 cm visual analogue scale (VAS) where each end of the scale was anchored by statements that defined the bounds of comfort from most comfortable to (most uncomfortable). Participants were asked to mark comfort levels on the line and the mark was assigned a score between 0 (i.e. most comfortable) and 100 (i.e. most uncomfortable), where 1 mm represented 1 point of the VAS score [33]. The VAS has been found to be a valid and reliable outcome measure for the measurement of pain and comfort levels in a wide range of samples [34, 35, 36].

### 2.4 Procedure

Data collection occurred at the UniSA podiatry clinic (City East Campus) where participants attended a single 1 h testing session. Participants were asked to avoid caffeine and exercise for 1 h prior to avoid influencing pressure measurements [37, 38]. Demographic data, medical history, bodyweight and height were obtained.

Attending in groups of one to three, participants were directed to testing rooms by an RA and asked to rest in supine, with feet and arms bare, on a clinic bed with their head elevated at approximately 20° for 10 min prior to the initial measurement [37, 39]. Automated measures of the left limb were taken once by a RA who did not require blinding as outcomes were calculated, displayed and stored via the automated unit. Automated measures were taken in the order of brachial, toe and thumb and occurred during the wait time between rater conducted measures. The RA also collected perceived comfort of the brachial and thumb measures once all testing was complete.

Raters were blinded to participants by a disposable curtain allowing only the right hand and foot of participants to be exposed and remained unaware of the identity of participants throughout the testing sessions. Raters measured the right limb only to avoid problems associated with statistical analysis of paired data and were required to leave a minimum of 10 min between measures for each participants [40]. Participants required a minimum of 3 min between measures to avoid reactive hyperaemia [37]. Noise, talking and participant movement was minimised [41] and room temperature controlled at 20–25°C to avoid influence on blood pressure measurements [20]. To reduce the wait time between measures for participants, Rater 1 (JM) consistently conducted the first and third manual measure, Rater 2 (HB) conducted the second and fourth manual measures. The order of measures (e.g. toe vs. thumb) were randomised as directed by computer generation for each rater [39].

**FIGURE 2:**
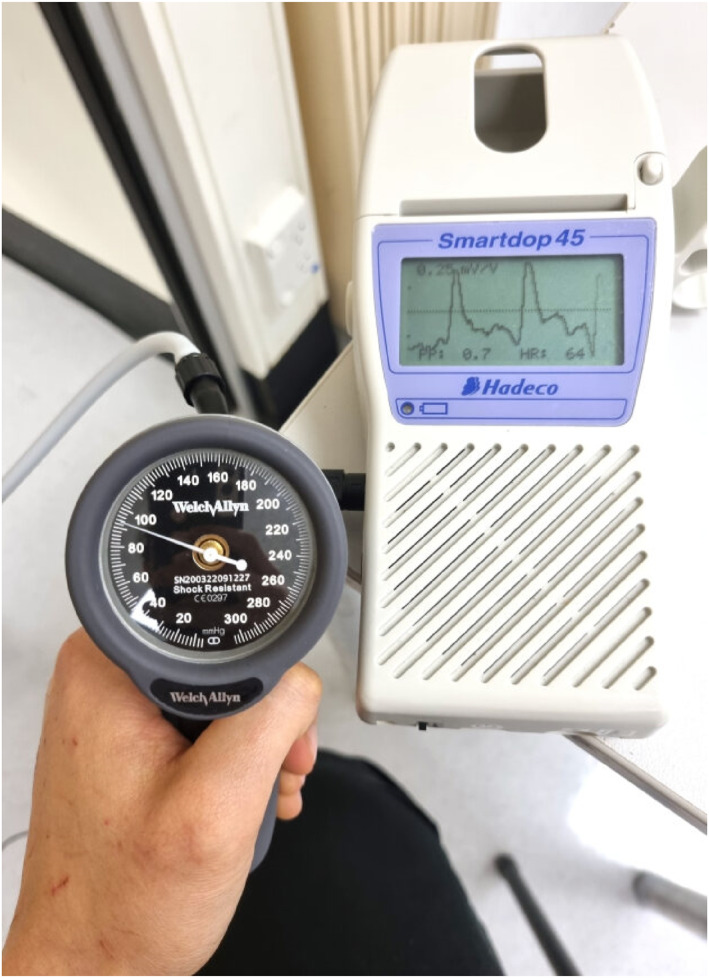
First return of pulse signal is recorded.

For ease of protocol timeline, Rater 1 (JM) took the first and third manual measure and Rater 2 (HAB) took the second and forth manual measure with order of measures randomised as directed by computer generation [42]. Blood pressures were taken as per recommended protocols [13, 15, 37], that is where the first return of pulse signal for each single measure was recorded (Figure 2). At the end of the session, both raters had collected two thumb and two toe pressures for each participant.

### 2.5 Data analysis

Participant characteristics are reported as frequencies and percentages. Data were assessed for normality via Shapiro–Wilk testing. Data were analysed using the Statistical Package Social Science software version 17.0 (SPSS Science) with significance level set at *p* ≤ 0.05. Based on an intraclass correlation coefficient (ICC) of 0.6, with a significance level of 0.05, a sample size calculation provided by G*power estimated a minimum of 22 participants for reliability outcomes [43].

Criterion validity for thumb and brachial pressures and toe–thumb index and TBI were calculated as Spearman correlation coefficients (rho). Scatter plots were used to identify if there was a relationship between measures. Spearman's correlation coefficients were interpreted by the strength of their relationship where values of 0.00–0.25 indicated a weak correlation, 0.26–0.51 fair, 0.51–0.76 moderate to good and 0.75–1.00 good to excellent [44]. The sign of the correlation indicated the direction (negative or positive) [44].

Concurrent validity between the automated and manual outcomes for the novel thumb pressure and TBI, and intra‐rater reliability between time points was explored using ICC [Model 3, 1] (two way mixed with absolute agreement) with 95% confidence intervals (95% CI), standard error of the mean (SEm), minimal detectable change (MDC). SEm reflects the reliability of a set of repeated measurements, a larger SEm indicates lower reliability. The MDC indicates the smallest amount of difference in a measure that ensures real change beyond random measurement error with 95% confidence [44]. SEm was calculated as: SEm = SD√(1 − *r*), and the MDC = 1.96 × SEm × √2 [45]. Bland Altman (BA) plots with 95% LOA provide a graphical display of the level of agreement for each rater between timepoints [46]. A priori decision was made to calculate SEm and MDC for the first time point for each rater to align more closely with clinical practice. Inter‐rater reliability was explored using ICC [Model 2, 2] 95% CI, SEm, MDC and BA Plots. ICC values were interpreted as >0.90 indicating excellent reliability, between 0.75 and 0.90 good reliability, between 0.5 and 0.75 moderate and <0.5 poor [44].

Results from the comfort scale were assessed for statistical significance with a paired *t*‐test and expressed as median and interquartile ranges [44].

Based on previous research [20], an a priori decision was made that measurement error should be lower than or equal to 20 mmHg to be clinically meaningful.

## 3 RESULTS

Thirty nine potential participants were screened, three were excluded based on inclusion criteria (outside of BMI range) and two due to equipment failure. Demographic and physical characteristics of the 34 participants who met the eligibility criteria are provided in Table 1.

**TABLE 1:** Participant demographic and physical characteristics (*N* = 39).

Characteristic	Median (IQR) unless otherwise noted
Gender	71.4% male (*n* = 21)
Age	23.0 years (range 19.0–42.0)
Height	170.5 cm (164.0–177.0)
Weight	71.3 kg (61.8–81.4)
BMI	24.5 (22.4–26.0)

Abbreviations: BMI, body mass index; IQR, interquartile range.

A total of 136 manual thumb and toe blood pressures and 34 automated brachial, thumb and toe blood pressures were included in the analyses.

### 3.1 Validity of measures

When compared to the reference standards (automated brachial measure and TBI respectively), fair to good correlations were observed for the novel thumb pressure outcomes.

**FIGURE 3:**
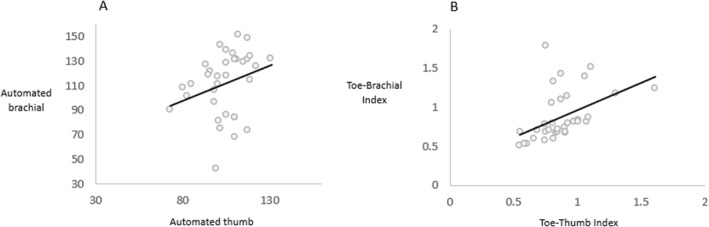
Scatterplot demonstrating (A) relationship between automated thumb blood pressure (*x*‐axis in mmHg) and brachial blood pressure (*y*‐axis in mmHg) measurements and (B) relationship between the toe–thumb index (*x*‐axis) and toe–brachial index (*y*‐axis).

A statistically significant fair positive correlation was observed between the automated thumb and brachial measures (*ρ* = 0.36, *p* = 0.03) (Table 2). This was confirmed by a scatter plot (Figure 3).

**TABLE 2:** Validity of automated thumb and brachial blood pressure measurements and toe–brachial index and toe–thumb index.

Measure	Median (IQR)	Rho (95% CI)	*p* value
Automated thumb	117.5 (89.0–131.0)	0.4 [100.2–120.5]	0.03
Automated brachial	105.00 (98.0–112.8)		
Toe‐brachial index	0.9 (0.8–1.0)	0.6 [0.8–1.0]	<0.00
Toe‐thumb index	0.8 (0.7–1.1)		

Abbreviations: 95% CI, 95% confidence interval; IQR, interquartile range; rho, Spearman correlation coefficients.

A statistically significant, good and positive correlation was observed between the calculated toe–thumb index and TBI (*ρ* = 0.62, *p* < 0.00) (Table 2), and confirmed visually by scatter plots (Figure 3).

### 3.2 Reliability of measures

Reliability of measures varied from poor to good (Tables 3 and 4).

**TABLE 3:** Intra‐rater reliability of measures.

Measure	Rater	Timepoint	ICC	95% CI	Mean (SD)	SEm (mmHg)	MDC (mmHg)	LOA (mmHg)
Thumb pressure (mmHg)	1	1 and 2	0.57	0.14, 0.79	117.54 (14.13)	2.68	7.43	−29.02 to 32.14
	2	1 and 2	0.74	0.49, 0.87	85.65 (23.01)	3.60	9.98	−44.44 to 37.85
Toe pressure (mmHg)	1	1 and 2	0.87	0.75, 0.94	87.54 (17.96)	2.10	5.79	−22.55 to 25.43
	2	1 and 2	0.53	0.07, 0.76	76.16 (10.86)	2.10	5.79	−29.05 to 50.64

Abbreviations: 95% CI, 95% confidence interval; ICC, intra‐class coefficient; LOA, levels of agreement; MDC, minimal detectable change; SD, standard deviation; SEm, standard error of the mean.

**TABLE 4:** Concurrent validity of manual and automated toe and thumb measures.

Measure	Rater	Timepoint	ICC	95% CI	Mean (SD)	SEm (mmHg)	MDC (mmHg)	LOA (mmHg)
Thumb pressure (mmHg)	1 and 2	1	0.16	−0.85, 0.47	101.16 (26.94)	4.52	12.53	−17.31 to 85.95
Toe pressure (mmHg)	1 and 2	1	0.16	−0.43, 0.54	82.87 (16.03)	3.49	9.67	−29.05 to 50.64
Manual versus automated—thumb pressure (mmHg)	1	1	−0.05	−1.06, 0.47	114.91 (20.86)	5.09	14.11	−64.99 to 51.35
	2	1	0.42	−0.16, 0.72	97.75 (9.21)	4.82	13.36	−27.62 to 82.62
Manual versus automated—toe pressure (mmHg)	1	1	0.69	0.39, 0.85	89.09 (19.22)	3.22	8.92	−35.18 to 38.47
	2	1	0.29	−0.22, 0.62	83.69 (16.96)	3.40	9.42	−21.41 to 26.65

Abbreviations: 95% CI, 95% confidence interval; ICC, intra‐class coefficient; LOA, levels of agreement; MDC, minimal detectable change; SD, standard deviation; SEm, standard error of the mean.

#### 3.2.1 Thumb pressures

**FIGURE 4:**
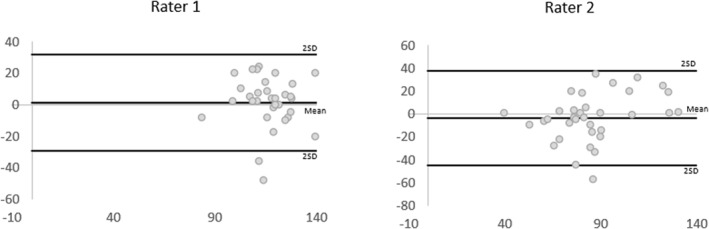
Bland–Altman plots displaying difference of measures (*y*‐axis in mmHg) against average of measures (*x*‐axis in mmHg) between timepoint 1 and 2 for thumb pressures. The lines represent the mean difference and the mean difference ± 2 SD. SD, standard deviation.

For intra‐rater reliability, outcomes were moderate for both raters (ICC [Model 3, 1] 0.57, 95% CI [0.14, 0.79] and ICC 0.74, 95% CI [0.49, 0.87] respectively) (Table 3). The BA plots (Figure 4) confirm the mean difference for both raters is close to zero, and the majority of data points lie within the LOA (2 SD of the mean). The SEm was consistently below 20 mmHg, indicating an acceptable mean measure error. The MDC outcomes indicate changes up to approximately 10 mmHg may be due to random error.

**FIGURE 5:**
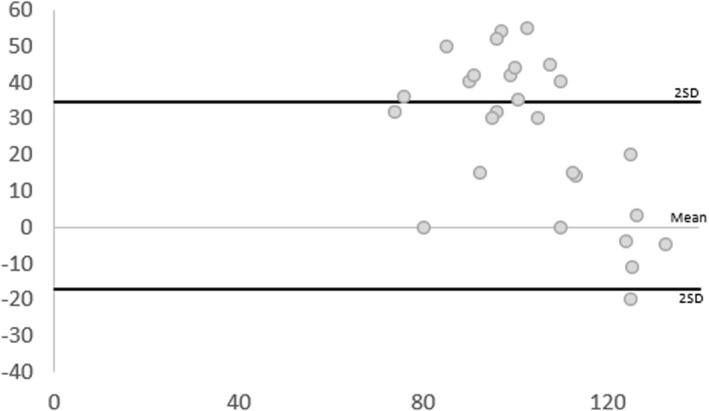
Bland–Altman plots displaying differences of measures (*y*‐axis in mmHg) and average of measures (*x*‐axis in mmHg) between Rater 1 and Rater 2 for thumb pressures. The lines represent the mean difference and the mean difference ± 2 SD. SD, standard deviation.

Inter‐rater reliability outcomes were poor with the 95% CI crossing zero (ICC [Model 2, 2] 0.16, 95% CI [−0.85, 0.47]) (Table 4). The BA plots illustrate a broad range of error from the mean difference and wide LOA (Figure 5). The SEm, MDC, and LOA for the inter‐rater measure were larger than for the intra‐rater reliability indicating greater error between raters than within raters.

#### 3.2.2 Toe pressures

**FIGURE 6:**
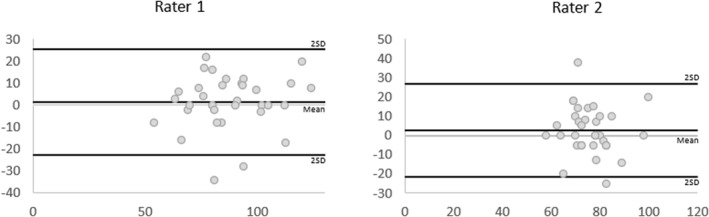
Bland–Altman plots displaying difference of measures (*y*‐axis in mmHg) against average of measures (*x*‐axis in mmHg) between timepoint 1 and 2 for toe pressures. The lines represent the mean difference and the mean difference ± 2 SD. SD, standard deviation.

Intra‐rater reliability outcomes were good and moderate for Rater 1 and 2 (ICC [Model 3, 1] 0.87, 95% CI [0.75, 0.94] and ICC 0.53, 95% CI [0.07, 0.76], respectively) (Table 3). As displayed in the BA plots (Figure 6), the spread of data for Rater 2 was wider and further from the mean difference than for Rater 1. The SEm, and MDC was identical for both raters.

**FIGURE 7:**
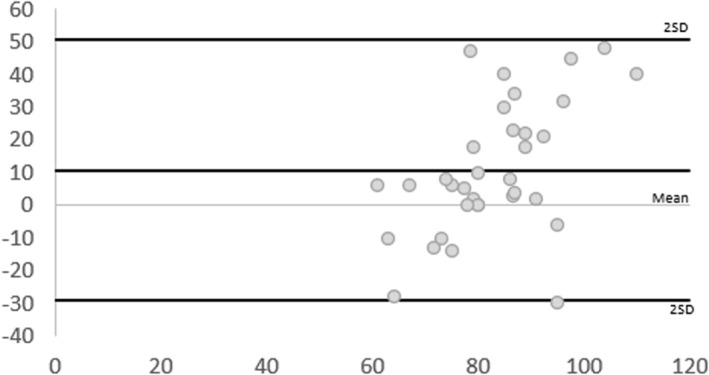
Bland–Altman plots displaying differences of measures (*y*‐axis in mmHg) and average of measures (*x*‐axis in mmHg) between Rater 1 and Rater 2 for toe pressures. The lines represent the mean difference and the mean difference ± 2 SD. SD, standard deviation.

Inter‐rater reliability of manual toe pressures was poor with 95% CI crossing zero, (ICC [Model 2, 2] 0.16, 95% CI [−0.43, 0.54)] (Table 4). Figure 7 illustrates the BA plots, displaying a broad range of error from the mean difference and wide LOA. Larger SEm, MDC and LOA for inter‐raters’ measures indicate greater reliability of measures within raters than between raters (Table 4).

#### 3.2.3 Manual versus automated measures

**FIGURE 8:**
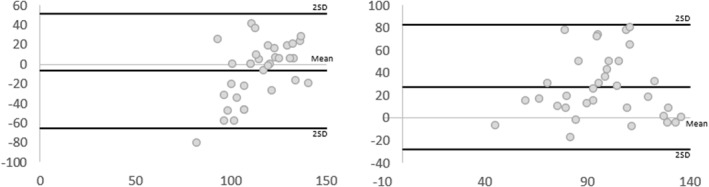
Bland–Altman plots displaying differences of measures (*y*‐axis in mmHg) and average of measures (*x*‐axis in mmHg) between raters and automated measures for thumb pressures. The lines represent the mean difference and the mean difference ± 2 SD. SD, standard deviation.

Concurrent validity for manual thumb pressure measures compared to the automated unit were poor and moderate respectively for Rater 1 and 2 with the 95% CI crossing zero for both raters (ICC [Model 3, 1] −0.05, 95% CI [−1.06, 0.72] and ICC 0.42, 95 CI [−0.16, 0.72], respectively) (Table 4). Lower SEm and MDC indicate greater reliability for Rater 2. The BA plots illustrate a wide LOA for both raters, with results from Rater 1 sitting further from the mean difference compared to Rater 2 (Figure 8).

**FIGURE 9:**
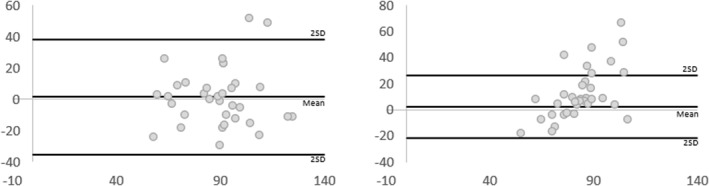
Bland–Altman plots displaying differences of measures (*y*‐axis in mmHg) and average of measures (*x*‐axis in mmHg) between raters and automated measures for toe pressures. The lines represent the mean difference and the mean difference ± 2 SD. SD, standard deviation.

Concurrent validity for toe pressure measures using the manual unit were moderate and poor for Rater 1 and 2 respectively when compared to automated outcomes with the 95% CI crossing zero for Rater 2 (ICC [Model 3, 1] 0.69, 95% CI [0.39, 0.85], ICC 0.29, 95% CI [−0.22, 0.62] respectively). SEm and MDC were lower for the toe than thumb indicating lower concerns regarding measurement errors (Table 4). The BA plots (Figure 9) illustrate a smaller LOA for both raters with results closer to the mean difference.

### 3.3 Perceived comfort of thumb and brachial measures

A paired samples *t*‐test found a significant difference between brachial and thumb comfort (median difference 5 mm VAS) (Table 5). The median score for participant comfort was 5/100 for thumb pressures and 10/100 for brachial measures, indicating thumb pressures were perceived as having higher levels of comfort. There was a greater variability for brachial comfort as noted by higher interquartile range.

**TABLE 5:** Outcomes of comfort scale.

Measure	Median (IQR)	*t* value	*p* value (95% CI)
Thumb comfort	5.0 mm (0.0–12.5)	3.39	<0.00 [3.1–12.2]
Brachial comfort	10.0 mm (2.0–32.5)		

Abbreviations: 95% CI, 95% confidence interval; median (IQR), median (interquartile range).

## 4 DISCUSSION

This study, to the best of our knowledge, is the first to explore the use of thumb pressures as an alternative to brachial pressures for determining correlations between upper and lower limb systolic pressures. Our findings suggest that thumb pressures only fairly correlated with brachial pressures, and the novel toe–thumb index had a good positive correlation with the current reference test of the TBI, however a large measurement error existed. Some positive outcomes were observed in terms of reliability and comfort for the novel thumb pressure measure and the proposed thumb–toe index. These outcomes suggest further investigations are warranted.

The findings of this study suggest (Table 2) thumb pressures and a toe–thumb index are potentially an alternative to brachial pressures and the TBI. However, whilst there was a good correlation between the TBI and toe–thumb index, as both used the same toe pressure measurements as a denominator, 50% of the numbers of the indices were the same. As expected, there was a better correlation between both indices then when the thumb and brachial was compared individually. Nevertheless, a pattern between automated thumb and brachial pressures was identified. The automated thumb systolic pressures from our study were consistently higher than automated brachial systolic pressures, supporting previous studies which have demonstrated non‐invasive wrist [47, 48] or finger [24, 49] pressures overestimate blood pressure in the upper arm by about 10 mmHg. This suggests with further investigations and the establishment of normative data, thumb pressures could be a comparable alternative to brachial pressures.

For inter‐rater reliability of manual thumb pressure, while MDC measures were all below our a priori value for acceptable error of 20 mmHg, the 95% LOA was largest, ranging from −17.31 to 85.95 (Table 4), suggesting a random measurement error well above our a priori value of 20 mmHg. Though there is a paucity a priori value for blood pressure measurement error, in the clinical context, this would be considered incredibly large. However, the interpretation of the thumb pressures findings should take into consideration the raters lack of experience with using manual measures.

The margin of error between raters for toe pressures in this study was better than the range of error for thumbs (Table 4). Both raters had greater experience with toe pressure measurements, which likely explains the smaller error for toes compared to thumbs. Additionally, the reasonable concurrent validity for Rater 2 when comparing manual and automated measures for thumb pressures suggests there is potential for thumb pressure measures to become more reproducible with adequate practice and training.

The ICC results for toe pressures observed in our study was generally lower than previous studies [19, 20]. However, wide margins of error, similar to our study, have been observed when measures are performed by the same or different raters [20, 50]. Manual TBI measures have also exhibited relatively wide LOA [20]. Considering toe pressures and the TBI are used clinically for diagnosis, this highlights the need for further investigation into the reliability of this measure and how much variation is considered acceptable within clinical practice.

Overall, participants perceived thumb measures as having higher levels of comfort than brachial. Perceived comfort being high for both measures highlights one possible advantage of using thumb pressures in clinical practice. Future investigations could assess the impact of the removal of clothes a source of discomfort or distress as well as assess practitioners' perceptions of the two measurement methods.

### 4.1 Limitations and further research

These findings should be interpreted in context of its limitations. The number of participants in sessions varied between 1 and 3. When only one participant was available, it prevented randomisation and blinding which may introduce bias. When two or more participants were present there was an increased interval of time between timepoints 1 and 2 for manual measures of up to an additional 10 min more creating a difference in consistency during the test/retest conditions. While unlikely in a healthy population, there may have been interlimb differences between left and right blood pressure measures. Though room temperature was controlled, it is possible the more prolonged resting times between timepoints caused changes in limb/skin temperature and digital vasoconstriction. Additionally, experience of using manual measures and the PPG unit between raters was minimal. Both the clinician in training and the experienced clinician had previously used the automated system, however, the manual system was only piloted prior to testing. This likely reduced reliability.

## 5 CONCLUSION

In conclusion, thumb systolic pressures are correlated with brachial systolic pressures, with moderate intra‐rater reliability for thumb pressures, however, correlation was only fair. Additionally, manual measurements did not correlate significantly with automated measurements and measurement error was wider than clinically acceptable. Consequently, caution is required in applying these techniques. As thumb measures were perceived as significantly more comfortable than brachial measures and have an advantage where brachial pressures cannot, or should not, be obtained, further evaluation is warranted. Future research on larger cohorts could evaluate the impact of training on concurrent validity of thumb pressures using manual and automated techniques and explore the validity of the use of thumb pressures as an alternative to brachial pressure measurements.

## AUTHOR CONTRIBUTIONS

**Juliana Mazzeo**: Conceptualization; methodology; investigation; data curation; visualisation; writing—original draft; writing—review and editing. **Helen A. Banwell** and **Kristin Graham**: Conceptualization; methodology; investigation; visualisation; writing—review and editing. **Peta E. Tehan**: Conceptualization; methodology; visualisation; writing—review and editing. **Grace Anderson**: Investigation; writing—review and editing.

## CONFLICT OF INTEREST STATEMENT

All authors declare that they have no competing interests.

## ETHICS STATEMENT

Approval was gained by the University of South Australia's Human Research Ethics Committee (Approval number 204110).

## CONSENT FOR PUBLICATION

Authors obtained written consent for participation and publication, including from participants in photographs.

## Data Availability

Raw data are available from authors on request.
